# Characterization of the inflammatory response of a canine intestinal epithelial cell line challenged with lipopolysaccharides and/or butyrate

**DOI:** 10.1093/jas/skaf404

**Published:** 2025-11-22

**Authors:** Francis D Phimister, David G Thomas, Neville Haack, Axel Heiser, Michelle J Farquhar, Emma N Bermingham, Rachel C Anderson

**Affiliations:** AgResearch Group, Bioeconomy Science Institute, Tennent Drive, Palmerston North, Manawatu, 4442, New Zealand; School of Agriculture and Environment, Massey University, Tennent Drive, Palmerston North, Manawatu, 4442, New Zealand; School of Agriculture and Environment, Massey University, Tennent Drive, Palmerston North, Manawatu, 4442, New Zealand; AgResearch Group, Bioeconomy Science Institute, Tennent Drive, Palmerston North, Manawatu, 4442, New Zealand; AgResearch Group, Bioeconomy Science Institute, Tennent Drive, Palmerston North, Manawatu, 4442, New Zealand; Waltham Petcare Science Institute, Leicestershire, LE14 4RT, UK; AgResearch Group, Bioeconomy Science Institute, Tennent Drive, Palmerston North, Manawatu, 4442, New Zealand; AgResearch Group, Bioeconomy Science Institute, Tennent Drive, Palmerston North, Manawatu, 4442, New Zealand

**Keywords:** cell model, cytokines, dog, intestinal barrier integrity, tight junctions

## Abstract

Cell culture models are used to assess the impacts that dietary changes have on host health; however, there is limited knowledge about canine intestinal cell models compared to those for humans. The objective of this study was to use a canine intestinal epithelial cell (cIEC) line to investigate responses to pro- and anti-inflammatory treatments relevant to health in the dog. It was anticipated that lipopolysaccharides (LPS) would cause a pro-inflammatory response, while butyrate would cause an anti-inflammatory response and mitigate the pro-inflammatory response induced by LPS. cIEC were stimulated with 250 µg/mL of LPS, 1 mM of butyrate, or a combination of LPS and butyrate compared to untreated controls over 8 h (*n* = 6 per treatment group). LPS treatment significantly reduced transepithelial electrical resistance (TEER) over time and caused increases in the protein abundance and gene expression of the pro-inflammatory interleukin (IL)-8 and chemokine (C-C motif) ligand (CCL)2 compared to untreated controls. Additionally, LPS treatment caused increases in the gene expression of the pro-inflammatory interferon gamma-induced protein (IP)-10, and increased protein abundances of keratinocyte chemotactic (KC)-like in apical and basal cell media compared to untreated cIEC. Butyrate failed to elicit an increase in anti-inflammatory cytokines, although treatment significantly reduced protein expression of the pro-inflammatory CCL2 in apical cell culture media and increased protein expression of IP-10. Butyrate reduced the LPS-induced IL-8 and KC-like increased pro-inflammatory protein abundances in basal cell culture media compared to untreated canine IEC and was found to reduce the LPS-induced intestinal barrier hyperpermeability. However, butyrate and LPS in combination caused an increase in CCL2 gene expression compared to treatment with butyrate and LPS alone. Therefore, this study has shown that butyrate can mitigate some aspects of the LPS-induced inflammatory response in an in vitro model of the canine intestine. Overall, changes in intestinal barrier integrity in response to the treatment were similar to that reported for human cell models; however, the molecular signaling somewhat differed, highlighting the need for further studies and illustrating the value of studying a canine cell model to understand the molecular responses in the dog.

## Teaser

Human cells are often used to model canine disease because of the lack of canine cell models. Here we show that the response of a canine intestinal epithelial cell model assessed here, to pro- and anti-inflammatory stimuli, is different to that of known human cell models, highlighting the need to further understand these species-specific differences and the value of using canine-specific cell models to understand molecular responses in the dog.

## Introduction

The intestinal epithelial barrier (IEB), formed by a single layer of intestinal epithelial cells (IECs), promotes and maintains host health in all animals, including dogs, by preventing unwanted components from entering the body and eliciting immune responses (Ahn et al., 2016; Assimakopoulos et al., 2018). Intestinal enterocytes, a specific class of IEC, are commonly used as a cell culture model to better understand intestinal barrier function (Snoeck et al., 2005; Peterson and Artis, 2014). These IECs form selectively permeable connections between cells, known as tight junctions (TJs) (Robinson et al., 2015; Vancamelbeke and Vermeire, 2017; Chelakkot et al., 2018) and express pattern recognition receptors (PRRs) that activate immune cascades upon the detection of pathogens or injured and damaged cells (Swerdlow et al., 2006; House et al., 2008; Zhang et al., 2010).

Several studies have investigated the use of IECs from dogs as models to assess the effects of diet on canine immune responses (Ramos-Vara and Miller, 2002; Weng et al., 2005; Golaz et al., 2007; Hemphill et al., 2009; Ohta et al., 2011; Farquhar et al., 2018; Reineking et al., 2018; Chandra et al., 2019). However, in these studies cell profiling was performed on limited snapshots of the immune response or changes to TJs. Without a wider overview of the responses to diet-based challenges in the dog, data from other models, such as humans, is often extrapolated to bridge gaps in understanding.

Dietary changes can significantly alter the composition of the microbiota in the gastrointestinal tract (GIT) of the dog (Allaway et al., 2020). The GIT microbiota assists the IECs in nutrient absorption, immune responses, and maintenance of IEB homeostasis (Belkaid and Hand, 2014; Blake and Suchodolski, 2016; Foster et al., 2017). Changes in microbiota composition leads to changes in abundance of microbial components and metabolites that can have either positive or negative effects on intestinal barrier function; however, again, most of this knowledge comes from studies using human cells.

Examples of beneficial microbial metabolites are short-chain fatty acids (SCFAs) (Peng et al., 2007; Bansal et al., 2010; Vinolo et al., 2011), including butyrate, acetate, and propionate, which modulate the IEC immune response and intestinal homeostasis in human models (Nery et al., 2012; Hang et al., 2013; Sandri et al., 2017). SCFAs activate T-cells and the production of cytokines through the G-protein coupled receptors 41 and 43 (Kim et al., 2013). Butyrate has been shown to increase barrier integrity in cells as assessed by TEER (Mariadason et al., 2001; Peng et al., 2009; Elamin et al., 2013; Feng et al., 2018), although high levels of butyrate (8 mM compared 2 mM) have been seen to paradoxically decrease TEER (Peng et al., 2007).

In contrast, lipopolysaccharides (LPS), which are part of the cell surface of Gram-negative bacteria, have consistently been found to reduce intestinal barrier integrity in human cell culture studies. LPS decreases TEER via reducing the expression levels of TJ proteins including zonula occludin-1 (Sheth et al., 2007; He et al., 2020), occludin (He et al., 2020; Wu et al., 2020), and claudin-1 (Fujita et al., 2012; Wu et al., 2020). Additionally, LPS activates the PRR Toll-like receptor (TLR)-4 and its subsequent downstream immune signaling cascades, which can also cause reduced TEER values and decreased intestinal barrier integrity (Ryu et al., 2017). Butyrate can downregulate the LPS-induced pro-inflammatory cytokine response of IECs and can alleviate the LPS-induced changes to zonula occludin-1 and occludin (Feng et al., 2018).

The objective of this study was to investigate the effects of butyrate and LPS on the inflammatory response of the canine intestine in vitro using a canine intestinal epithelial cell (cIEC) line (Farquhar et al., 2018). These cIEC were previously isolated canine small intestinal tissue (Weng et al., 2005) and immortalized using a temperature-sensitive mutant of the SV40 T-Ag (Quaroni and Beaulieu, 1997). The expected outcomes were that: 1) LPS alone would cause a pro-inflammatory response, reduce protein abundances of TJ proteins associated with barrier function, and increase barrier permeability; 2) Butyrate alone would cause an anti-inflammatory response, increase protein abundances of TJ proteins associated with ­barrier function, and reduce barrier permeability; and 3) Butyrate would mitigate the pro-inflammatory response induced by LPS. cIEC were treated with LPS, butyrate or a combination of the two, with untreated cIEC in parallel as a control. The effects were quantified using TEER to assess barrier integrity of the cIEC, Nanostring to profile the changes in gene expression of the cIEC, and Luminex to examine the changes in cytokine protein expression.

## Materials and Methods

### Cell maintenance

The canine intestinal epithelial single clonal cell line (cIEC) was supplied by Waltham Petcare Science Institute (UK) and cultured as previously described (Farquhar et al., 2018). cIEC were maintained in cell culture flasks and propagated in Opti-Minimal Essential Medium I Reduced Serum Media (Opti-MEM; ThermoFisher; Carlsbad, California, USA) supplemented with 4% foetal bovine serum (FBS; ThermoFisher), 2 mM L-glutamine (ThermoFisher), 2 mM GlutaMAX Supplement (ThermoFisher), 10 mM N-2-hydroxyethylpiperazine-N-2-ethane sulphonic acid (HEPES; ThermoFisher), 20 ng/mL epidermal growth factor (EGF; Sigma Aldrich; St Louis, Missouri, USA), 10 µg/mL insulin (Sigma), and 150 nM hydrocortisone (Sigma), hereafter referred to as Growth Medium, at 32°C, 6% v/v CO_2_. Cells were passaged at 80% confluence using TrypLE™ (ThermoFisher).

### Cell differentiation

cIEC were seeded onto 24-well inserts at a density of 2.7 × 10^5^ cells/cm^2^ in Growth Medium. These were cultured for 24 h at 32°C, 6% v/v CO_2_. After 24 h, the culture medium was removed and the cIEC were replenished with Dulbecco’s Modified Eagle Media (DMEM) supplemented with 7% FBS, 2 mM L-glutamine, 2 mM GlutaMAX, 10 mM HEPES, and 150 nM hydrocortisone, hereafter referred to as Differentiation Medium. These cells were then cultured for 48 h at 39°C, 6% v/v CO_2_.

### Cell stimulation

After 48 h in Differentiation Medium, the apical media in the insert was replaced with the treatment (*n* = 6 per treatment). The Control Medium was DMEM supplemented with 7% FBS, 2 mM L-glutamine, 2 mM GlutaMAX, 10 mM HEPES. The other treatment groups contained either 250 µg/mL LPS, 1 mM butyrate, or 250 µg/mL LPS and 1 mM butyrate (Combination) in Control Medium. The cells were incubated for 8 h at 39°C, 6% v/v CO_2_. The LPS and butyrate doses and the treatment duration were chosen based on previous experiments (Phimister, 2023).

### TEER measurement

To quantify TEER, the resistance across the cIEC layer was measured using an EndOhm TEER cup (World Precision Instruments, Sarasota, Florida, USA) connected to an EVOM voltohmmeter (World Precision Instruments, Sarasota, Florida, USA) every 2 h for 8 h. The inserts containing the differentiated cIEC monolayers were transferred from the insert plate to the TEER cup containing Control medium preheated to 39°C. Resistance was immediately measured, without equilibration to room temperature, and converted to TEER (Ω and Ωcm^2^, respectively) using equation 1. To evaluate the impacts of the LPS and butyrate challenges on TEER, data were expressed as a percentage change in TEER over time calculated using equation 2. The ‘initial TEER’ is the TEER prior to addition of the treatment, and the ‘current TEER’ is the TEER value at the given timepoint post addition of the treatment.

Equation 1. *Calculation of TEER from raw Ohmic resistance*


$$\text{TEER}\;\left(Ω{\text{cm}}^{2}\right)=\text{Resistance}\;\left(Ω\right)\times \text{membrane}\;\text{area}\;\left({\text{cm}}^{2}\right)$$

where the membrane area was 0.3 cm^2^

Equation 2. *Calculation of the change in TEER, expressed as a percentage of the original TEER reading*


$$\text{Change}\;\text{in}\;\text{TEER}\;\left(\%\right)=\frac{\left({\text{TEER}}_{\text{current}}–{\;\text{TEER}}_{\text{initial}}\right)}{{\text{TEER}}_{\text{initial}}}\times 100.$$

### Gene expression analysis

After 8 h of treatment, cells were dissociated from the apical surface of the insert using TryPLE™. Harvested cells were centrifuged for 2 min at 300 × *g*, the cell pellet re-suspended in 30 µl of Qiagen RLT buffer and frozen at −80°C. The lysed cIEC were subsequently prepared for mRNA analysis using a QIAGEN Micro Kit, following manufacturer instructions. Gene expression analysis was performed using the nCounter Analysis system (NanoString Technologies Inc., Seattle, WA), as described elsewhere (Quirke et al., 2021). The list of 83 gene targets is included as Supplementary Table S1. This included five housekeeping genes previously observed as having highly abundant mRNA expression in the canine intestine (Cho et al., 2013). Two samples from each treatment group were combined to allow sufficient RNA content for expression analysis and were analyzed once.

### Protein abundance analysis

Cell media was collected at the end of the 8-h period of treatment and immediately stored -80°C until assayed. Protein abundance analysis was performed by the Massey Nutrition Laboratory using the MILLIPLEX^®^ Canine Cytokine/Chemokine Magnetic Bead Panel—Immunology Multiplex Assay (Merck, Rahway, New Jersey, USA) as per manufacturer instructions. The list of the 13 proteins quantified is given in Supplementary Table S2. All samples were tested in duplicate.

### Statistical analysis

The statistical analyses for the TEER data were performed using R (R Core Team, 2020). The effect of treatment on change in TEER over time was compared using a repeated measures mixed-effects model to account for the fact that the same cell monolayers were measured over time. Models were fitted by the maximum-likelihood method using the *nlme* package (Pinheiro, 2022) in R. The statistical model included the effect of treatment, time, and their interaction as fixed effects, and the Transwell inserts nested within blocks (where one run of an experiment was considered a block) as a random effect. Post-hoc pairwise comparisons with the Bonferroni correction were applied to the estimated marginal means using the *emmeans* package (Lenth, 2022). The false discovery rate (q) was applied to the tests of the marginal means, with differences considered significant when *q* < 0.05.

For the statistical analysis of the gene expression and protein abundance results Genstat (19th Edition, VSN International, Hemel Hempstead, UK) was used. MANOVAs were also performed on the nanostring RNA counts and to assess the differences in treatments and sample locations for Luminex results. When the MANOVA indicated that there were treatment effects, then a pairwise comparison with Bonferroni post-hoc corrections was performed. Data visualization was performed using Genstat. Statistical significance is reported as a *P* value < 0.05, whilst *P *< 0.1 was considered a trend. All data are presented as mean ± SEM unless otherwise noted.

## Results

### Butyrate alleviated the LPS-induced decreases in TEER

**Figure 1.**
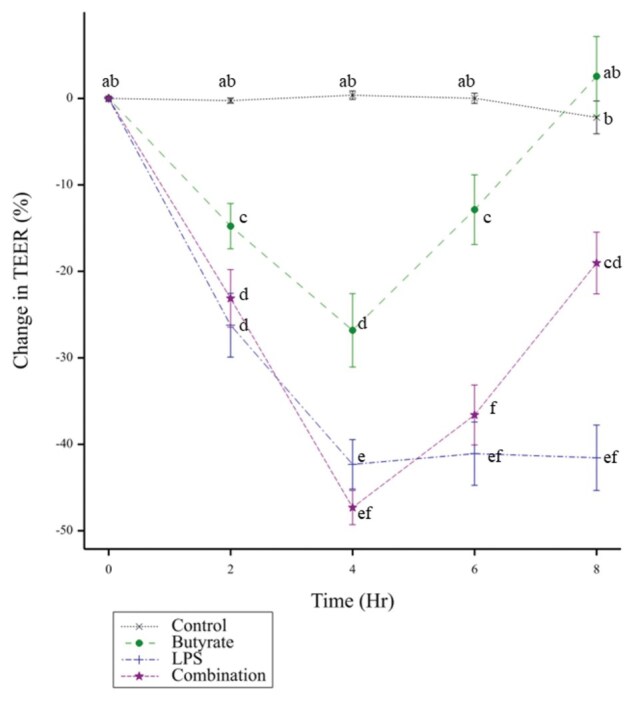
Mean (±SEM) change in TEER from cIEC incubated with different treatments. Change in TEER was defined as a percentage change compared to the initial, time 0 value for the same treatment. Treatments were 1 mM of sodium butyrate (Butyrate), 250 μg/mL lipopolysaccharides (LPS), both 1 mM sodium butyrate and 250 μg/mL LPS (Combination), and untreated cIEC (Control). *N* = 6 for each treatment. Timepoints that do not share a common letter denotes a significant (*P* < 0.05) difference in the change in TEER.

Changes to barrier integrity of the cIEC caused by stimulation with LPS, butyrate, or a combination of the two, were calculated as a change in TEER (Figure 1). TEER was significantly impacted by treatment over time, an effect not observed in the untreated cIEC. Butyrate caused an initial decrease in TEER at 2–6 h post-treatment compared to 0 h and recovered after 8 h. At 8 h post-treatment, cIEC treated with LPS alone had the greatest reduction in TEER significantly lower compared to all other treatments. Combination of butyrate and LPS demonstrated that butyrate alleviated the LPS-induced decrease to TEER. However, the TEER was still lower than that of the untreated and butyrate-treated cIEC.

### Expression of CCL2/MCP-1, IL-8, and IP10 genes was increased by LPS stimulation

**Figure 2.**
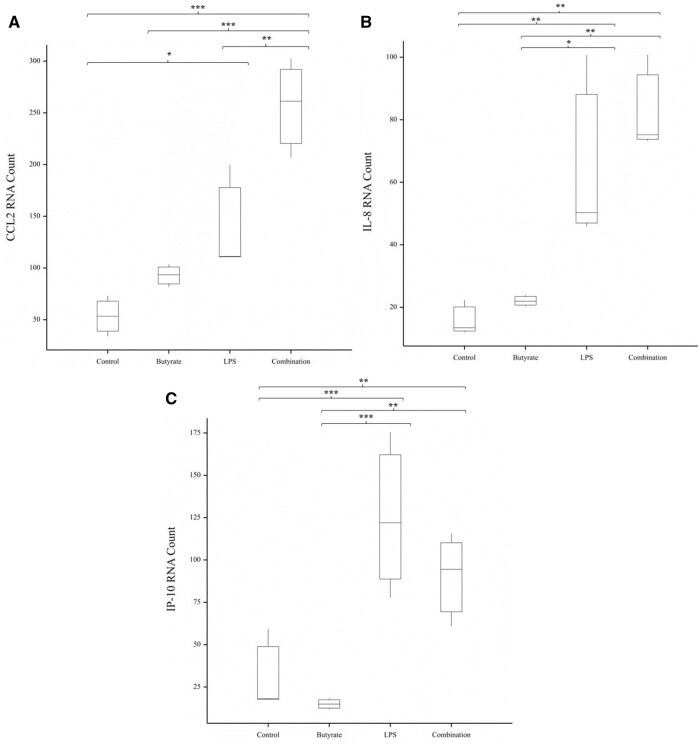
Genes that were significantly differentially expressed between cIEC that were untreated (Control) or treated with 1 mM of sodium butyrate (Butyrate), 250 μg/mL lipopolysaccharides (LPS) or both 1 mM sodium butyrate and 250 μg/mL LPS (Combination) for 8 h. Differently expressed genes were (A) CCL2, (B) IL-8 and (C) IP-10. *N* = 3 for each treatment. Significant differences are represented by *, where * *P* < 0.05, ** *P* < 0.01, *** *P* < 0.001.

The effects of butyrate and LPS alone and in combination on cIEC gene expression after 8 h were assessed. Of the 78 target genes, the three genes shown in Figure 2 were differentially expressed between treatment groups. Treatment with LPS, or a combination of LPS and butyrate caused a significant increase in the gene expression levels of chemokine (C-C motif) ligand 2 (CCL2; also known as monocyte chemoattractant protein (MCP)-1), interleukin (IL)-8 and IP-10 compared to the untreated controls and those treated with butyrate alone. Gene expression was not different between the control or butyrate treated cIEC. Gene expression with non-significant differences between treatments data are included in Supplementary Table S1.

### Abundance of IL-8, IP10, KC-like, and MCP-1/CCL2 proteins was increased by LPS stimulation

**Figure 3.**
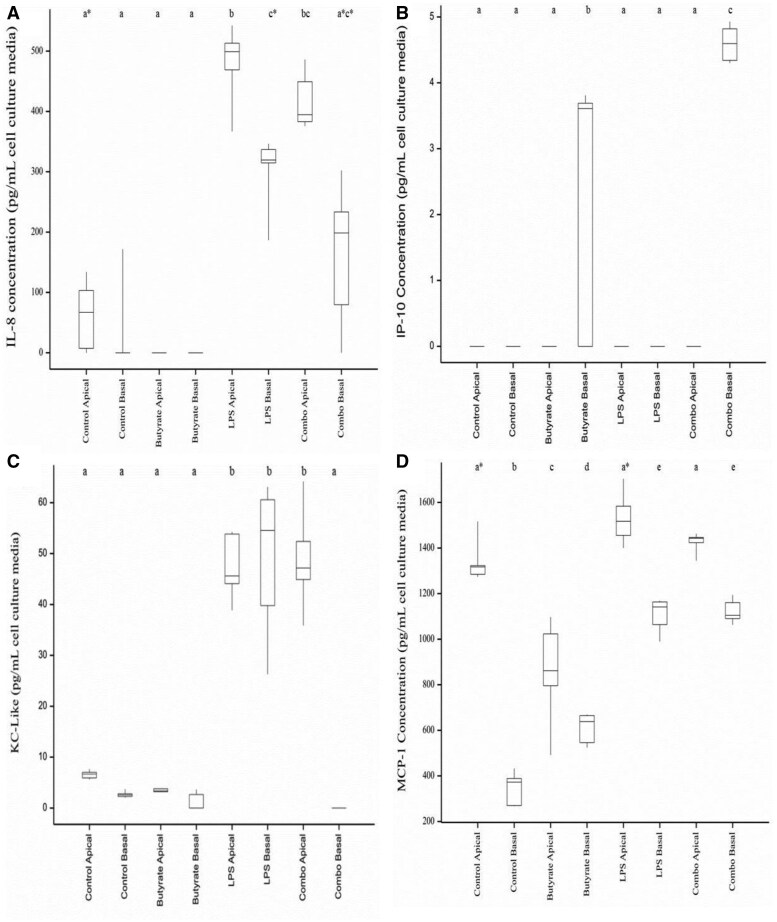
Proteins that were significantly differentially abundant between cIEC that were untreated (Control) or treated with 1 mM of sodium butyrate (Butyrate), 250 μg/mL lipopolysaccharides (LPS) or both 1 mM sodium butyrate and 250 μg/mL LPS (Combination) for 8 h. Differentially abundant proteins were (A) IL-8, (B) IP-10, (C) KL-like and (D) MPC-1. N = 6 for each treatment and location. Significant differences are represented by *, where * *P* < 0.05, ** *P* < 0.01, *** *P* < 0.001.

Following treatment with LPS, butyrate or combination of, media was harvested from the apical and basal cIEC culture chambers and cytokine and chemokine concentrations quantified. Of the 13 proteins assessed, the four shown in Figure 3 were differentially abundant between treatment groups and sample location. Cells exposed to LPS had higher abundance of KC-like and IL-8 in both the apical and basal media compared to untreated controls. This was reduced in the cells treated with both LPS and butyrate in the basal media only. The cells treated with butyrate had higher abundance of IP-10 in the basal media, which was further increased in the combination treatment, compared to untreated and LPS only treated cells. For MCP-1/CCL2, compared to untreated controls, LPS and combination treatment increase abundance in the basal, but not apical media; whereas treatment with butyrate, reduced abundance in apical media and increased abundance less than LPS and combination treatment. For the proteins that were not differentially abundant between treatment groups data is included in Supplementary Table S2.

## Discussion

This study measured the inflammatory response of cIEC to LPS and butyrate in the canine gut by assessing the impacts across cytokine response, TJ expression, and barrier integrity *in vitro*, and is the first study that has assessed in parallel both protein abundances and gene expression of the immune cascades and TJs in a canine cell model. In line with our first hypothesis, LPS treatment increased pro-inflammatory IL-8 cytokine protein abundance and gene expression and reduced TEER over time; however, there were no changes in TJ gene expression. There was limited evidence to support our second hypothesis because although butyrate treatment reduced the cytokine concentration of pro-inflammatory CCL2 in apical cell culture media, there was no improvement in TEER or increase in TJ gene expression from butyrate treatment. Finally, though stimulation with only butyrate did not alter TEER, there were reduced and restored LPS-induced increases to intestinal barrier permeability, supporting the third hypothesis. The effect of butyrate and LPS together on cytokine protein abundance and gene expression were mixed. Butyrate reduced LPS-induced increases to pro-inflammatory IL-8 and KC-like cytokine concentrations in basal cell culture media, but butyrate and LPS together caused an increase in CCL2 expression compared to treatment with butyrate and LPS alone.

A key driver for this study was to compare the response of cIEC to the known responses of human IEC models, such as Caco-2 cells, to identify canine-specific responses and justify the use of cIEC when studying outcomes for dogs. This was both due to the lack of relevant in vivo studies in the dog to compare against but also to emphasize the differences in species response and use these as foundations for future knowledge in the dog. In relation to barrier integrity, as measured by the TEER assays, the cIEC largely behaved similarly to human IEC. For example, the TEER in cIEC monolayers was significantly altered over time by LPS treatment, in agreement with IEC models from other species (Hanson et al., 2011; Stephens and von der Weid, 2020). In studies with human cells a paradoxical effect of butyrate has been observed, wherein low concentrations (2 mM) improve TEER or maintain TEER stability, and high concentrations (8 mM) reduce it (Gibson et al., 1999; Peng et al., 2007; Elamin et al., 2013). In this study the butyrate treatment did not cause an improvement to TEER over time, although the time window in this study was only 8 h, whereas in Caco-2 cells the improvement from butyrate treatment was seen after 24 h, and up to 96 h (Peng et al., 2007). The timeframe for this study was chosen based on the maximum production of IL-8 in preliminary studies (Phimister, 2023), not based on TEER changes. The observed pattern of response in TEER to the butyrate challenge of cIEC suggests the potential to reproduce the effect in human IECs over a longer period. Additionally, this study showed that butyrate was able to reduce and restore LPS-induced disruptions to intestinal barrier permeability, which is also seen in human cell studies (Yan and Ajuwon, 2017).

In contrast, the patterns of expression of TJ genes and proteins in response to the treatments differed in the canine cells compared to that established in human cells. In human cell studies, changes in TEER are generally observed in tandem with changes to TJ gene expression and protein abundances, wherein claudin-2 expression tends to decrease, and increases are seen in claudins -1, -3, and -4, in addition to occludin and zonula occludin-1 (Sheth et al., 2007; Fujita et al., 2012; Yan and Ajuwon, 2017; He et al., 2020; Wu et al., 2020). However, this was not observed in this study. Similarly, the increases in TLR2 and TLR4 to mitigate LPS-induced changes on TEER (Hanson et al., 2011; Stephens and von der Weid, 2020) were also not observed in this study. There was detectable gene expression from these targets across treatments in this study; however, none were altered by treatment. Further investigations with other dog IEC will determine if these results are inherent to the canine intestine, or if this is a trait unique to this cIEC model.

Differences in responses to the treatments in terms of cytokine expression in the canine cells compared to those reported for human cell lines were also observed. One example is IL-8, a pro-inflammatory cytokine that can act as an attractant and infiltrator of neutrophils and is commonly raised in intestinal diseases such as inflammatory bowel disease (Fusunyan et al., 1998; Asarat et al., 2015). In this study, IL-8 gene expression and cytokine content were increased in cIEC challenged with LPS, which is consistent with cell culture studies in other species (Angrisano et al., 2010; Kainulainen et al., 2015). In contrast, butyrate also increases IL-8 gene expression in human HT-29 and Caco-2 cells (Fusunyan et al., 1998; Asarat et al., 2015), but there was no increase in IL-8 gene expression or cytokine concentration in the cIEC. Butyrate reduces LPS-induced IL-8 cytokine concentrations in human endothelial cells (Li et al., 2018), and here we similarly demonstrated butyrate reduced LPS-induced IL-8 cytokine concentrations in the basal cell media only. Apical secretions of IL-8 are suggested to demonstrate luminal autocrine functionality (Rossi et al., 2013); whereas basal secretions recruit neutrophils to sites of infection and injury (Rossi et al., 2013). Therefore, the decreased IL-8 concentrations in basal media from butyrate-stimulated cIEC suggest butyrate reduces the pro-inflammatory pathways in the host. These results show that the cIEC responds similarly to IEC from other species; however, there are some differences which could be species-specific. Additional research using other IECs from dogs and other species are required to confirm the source of these differences.

For IP-10, differences were also seen in its expression patterns in the cIEC model compared to published studies with human cell lines. IP-10 is secreted in response to interferon-γ and chemotactically attracts T-cells, monocytes, and dendritic cells (Chen et al., 2020); however, differences in IP-10 cytokine concentrations have yet to be observed in canine serum (Calvalido et al., 2016; Galán et al., 2018), plasma (Mazrier et al., 2022), or spinal fluid (Taylor et al., 2014) between healthy and unhealthy individuals. In this study, the gene expression of IP-10 was increased, albeit in the absence of changes in protein expression, in cIEC treated with LPS. In comparison, IP-10 cytokine concentrations were found to increase in human blood monocytes (Di Lorenzo et al., 2020) and rat microglia (Mayer et al., 2016) in response to LPS. This limited stimulation of IP-10 production by LPS in the canine cells may conform with what occurs in vivo.

In contrast, for CCL2, which is an inflammatory chemokine that attracts and activates macrophages and basophils (Carson et al., 2017) via TLR4 signaling (Martin-Vaquero et al., 2014), the expression pattern in the dog cells aligned to that reported for human cells. The gene expression and concentration in apical cell media were increased in cIEC treated with LPS, consistent with results seen in human monocytes (Akhter et al., 2018). In human IECs, butyrate decreases the expression of CCL2 (Fusunyan et al., 1999). Interestingly, though butyrate treatment of the cIEC decreased cytokine concentration of CCL2 in the apical media compared to the untreated control, there was no difference observed in the gene expression between the two treatments. Further work could assess the changes in gene expression at earlier timepoints to determine if the lack of difference observed in this experiment was due to time.

Similarly, the expression of KC-Like (also called chemokine (C-X-C motif) ligand 1 (CXCL-1)) was similar in cIEC and human IEC. KC-like levels were increased in both the apical and basal cell culture media of cIEC treated with LPS and in the apical media of cIEC treated with LPS and butyrate. There was no change in relation to the untreated control amongst all other treatments and cell media location. KC-Like is suggested to be a potential biomarker for sepsis in dogs (Karlsson et al., 2016; Goggs and Letendre, 2019). LPS-induced sepsis has been modeled in human Caco-2 cells (Ling et al., 2016; Wei et al., 2022). Thus, the results obtained here suggest the potential use of the cIEC model in sepsis modeling.

Interestingly, there was no indication of an anti-inflammatory effect of butyrate in the cIEC. IL-10 is one of the key anti-inflammatory cytokines involved in immune response (Couper et al., 2008) and has been detected the colonic mucosa of dogs (Peters et al., 2005; Tamura et al., 2014). However, there was no detectable IL-10 in this study suggesting that the cIEC do not mount an anti-inflammatory IL-10 response to expected stimuli. This is an important consideration prior to future investigations using the cIEC as a model of inflammation in assumptions regarding the inflammatory response in the canine intestine.

This study was limited in that there were no other cell lines provided for comparison, due to a lack of available species appropriate cell lines. As such, it remains unclear if the results presented, namely the limited number of significantly altered genes/proteins, are indicative of a response that would be observed in the dog, or artifacts of this cell line, as such additional studies to explore the effect of increased treatment times are recommended. Despite these limitations, these results provide an initial dataset for use as a baseline in future studies that would be beneficial for understanding in the literature, using of these cells as a model of the dog intestine.

This study showed that this cIEC model behaves similarly to human cell culture models in terms of its barrier integrity response. However, the observed gene and protein expression from bacterial and SCFA stimuli differs. This illustrates that it is important to use a canine cell model to mimic responses that are specific to the dog. Future work building on the concepts established here could begin by looking at other SCFAs and bacterial ligands to build a better picture of how the GIT microbiota and diet-derived SCFA impact the intestinal health of the dog. Additionally, the use of another dog IEC to compare these results against would further shed light on potential species-derived differences in intestinal responses.

## Supplementary Material

skaf404_Supplementary_Data

## Abbreviations:

cIEC: canine intestinal epithelial cell
CCL: chemokine (C-C motif) ligand
DMEM: Dulbecco’s modified eagle media
EGF: epidermal growth factor
FBS: foetal bovine serum
GIT: gastrointestinal tract
HEPES: N-2-hydroxyethylpiperazine-N-2-ethane sulphonic acid
IEB: intestinal epithelial barrier
IEC: intestinal epithelial cell
IL: interleukin
IP10: interferon gamma-induced protein 10
KC-Like: keratinocyte chemotactic-like
LPS: lipopolysaccharides
MCP: monocyte chemoattractant protein
mRNA: messenger ribonucleic acid
OPTI-MEM: Opti-Minimal Essential Medium I Reduced Serum Media
PBS: ­phosphate buffered saline
PRR: pattern recognition receptor
RNA: ribonucleic acid
SCFA: short-chain fatty acid
TEER: transepithelial electrical resistance
TLR: Toll-like receptor
TJ: tight junction
