# Supplementary material for: Characterization of the inflammatory response of a canine intestinal epithelial cell line challenged with lipopolysaccharides and/or butyrate
Source: J Anim Sci. 2025 Nov 22;104:skaf404. doi: 10.1093/jas/skaf404 (PMC12863941; doi:10.1093/jas/skaf404)
Supplement: skaf404_Supplementary_Data [file skaf404_supplementary_data.docx]

**Supplementary Table S1:** RNA counts from Nanostring analysis of cIEC collected 8 hours post-challenge with different treatments. Each result is presented as mean ± SEM. Each treatment comprised n = 3, and the impacts of treatment presented as the p value. Results that do not share the same superscript letter are significantly different in RNA counts (p < 0.05). Significant p values and associated gene targets are listed in bold.

| **Gene** | **Description** | **NCBI Gene ID** | **Control** | **Butyrate** | | **LPS** | **Combination** | | **P value** | |
| --- | --- | --- | --- | --- | --- | --- | --- | --- | --- | --- |
| ***Target Genes*** | | |  |  |  |  |  |  |  |  |
| *AJAP1* | Adherens junctions associated protein 1 | 607839 | 0 ± 0 | 0 ± 0 | | 0 ± 0 | 0 ± 0 | | N/A | |
| *CASP4* | Caspase 4, apoptosis-related cysteine peptidase | 403724 | 8 ± 4.1 | 8.7 ± 4.4 | | 0 ± 0 | 0 ± 0 | | 0.124 | |
| *CBD103* | Beta-defensin 103 | 100170103 | 3.5 ± 3.5 | 5.6 ± 5.6 | | 0 ± 0 | 0 ± 0 | | 0.579 | |
| ***CCL2*** | **C-C motif chemokine ligand 2** | **403981** | **53.4 ± 11.1^a^** | **92.8 ± 6.2^a^** | | **140.5 ± 29.6^b^** | **256.7 ± 27.7^c^** | | **<0.001** | |
| *CCL3* | C-C motif chemokine ligand 3 | 448787 | 9.9 ± 5.4 | 10.6 ± 5.4 | | 6.2 ± 6.2 | 19.2 ± 2.3 | | 0.381 | |
| *CCL4* | C-C motif chemokine ligand 4 | 448786 | 4 ± 4 | 0 ± 0 | | 0 ± 0 | 0 ± 0 | | 0.441 | |
| *CCL7* | C-C motif chemokine ligand 7 | 491148 | 14.7 ± 3.4 | 17.2 ± 2.9 | | 19.7 ± 3.4 | 20.9 ± 1.3 | | 0.484 | |
| *CD4* | CD4 molecule | 403931 | 4 ± 4 | 0 ± 0 | | 3.8 ± 3.8 | 3.7 ± 3.7 | | 0.801 | |
| *CD14* | CD14 molecule | 607076 | 0 ± 0 | 4.2 ± 4.2 | | 0 ± 0 | 0 ± 0 | | 0.441 | |
| *CDH1* | Cadherin 1, type 1, E-cadherin (epithelial) | 442858 | 86.2 ± 22.2 | 52.5 ± 5.5 | | 73.3 ± 29.6 | 75.9 ± 7.7 | | 0.666 | |
| *CLDN1* | Claudin 1 | 608207 | 4.9 ± 4.9 | 4 ± 4 | | 3.8 ± 3.8 | 16.9 ± 4.5 | | 0.178 | |
| *CLDN10* | Claudin 10 | 476963 | 0 ± 0 | 0 ± 0 | | 0 ± 0 | 0 ± 0 | | N/A | |
| *CLDN11* | Claudin 11 | 488160 | 13.4 ± 1.2 | 8.7 ± 4.4 | | 10.3 ± 5.8 | 8.6 ± 8.6 | | 0.923 | |
| *CLDN12* | Claudin 12 | 608397 | 4 ± 4 | 3.9 ± 3.9 | | 7.7 ± 3.8 | 4.8 ± 4.8 | | 0.908 | |
| *CLDN14* | Claudin 14 | 487751 | 0 ± 0 | 0 ± 0 | | 0 ± 0 | 0 ± 0 | | N/A | |
| *CLDN15* | Claudin 15 | 608226 | 31 ± 6.2 | 24.2 ± 5.1 | | 27.2 ± 7.6 | 24.8 ± 3.3 | | 0.839 | |
| *CLDN16* | Claudin 16 | 608218 | 3.5 ± 3.5 | 7.4 ± 3.7 | | 5.2 ± 5.2 | 8.6 ± 4.4 | | 0.844 | |
| *CLDN17* | Claudin 17 | 487720 | 0 ± 0 | 0 ± 0 | | 11 ± 6.2 | 9.7 ± 4.9 | | 0.154 | |
| *CLDN18* | Claudin 18 | 477079 | 0 ± 0 | 7.3 ± 3.6 | | 4.3 ± 4.3 | 13.2 ± 1 | | 0.061 | |
| *CLDN19* | Claudin 19 | 607005 | 33.2 ± 4.9 | 43.9 ± 6.9 | | 32.5 ± 4.5 | 35.9 ± 8.8 | | 0.62 | |
| *CLDN2* | Claudin 2 | 403649 | 12 ± 0.6 | 12.2 ± 6.3 | | 13.6 ± 2.5 | 15.8 ± 2.3 | | 0.866 | |
| *CLDN3* | Claudin 3 | 403648 | 37.3 ± 10.1 | 28.9 ± 5.7 | | 27.8 ± 5.6 | 39 ± 6.5 | | 0.618 | |
| *CLDN4* | Claudin 4 | 100856416 | 5.3 ± 5.3 | 14 ± 1.8 | | 12.8 ± 0.7 | 9.1 ± 4.5 | | 0.396 | |
| *CLDN5* | Claudin 5 | 100684266 | 28.5 ± 2.1 | 26.4 ± 3.7 | | 29.1 ± 4.1 | 36.2 ± 7.8 | | 0.559 | |
| *CLDN6* | Claudin 6 | 490048 | 32 ± 8.3 | 28.2 ± 2.8 | | 32.7 ± 6.6 | 41.4 ± 7.2 | | 0.564 | |
| *CLDN7* | Claudin 7 | 489466 | 74.4 ± 17.1 | 51.8 ± 6.5 | | 55.8 ± 19.4 | 73.8 ± 2.8 | | 0.54 | |
| *CLDN8* | Claudin 8 | 478401 | 18.5 ± 5.5 | 11 ± 5.5 | | 19.4 ± 0.4 | 20.7 ± 3.4 | | 0.428 | |
| *CLDN9* | Claudin 9 | 490049 | 13.9 ± 2.5 | 13.5 ± 1.4 | | 12.2 ± 6.1 | 12.8 ± 0.9 | | 0.985 | |
| *CRP* | C-reactive protein | 488629 | 0 ± 0 | 0 ± 0 | | 0 ± 0 | 4.5 ± 4.5 | | 0.441 | |
| ***CXCL8*** | **Chemokine (C-X-C motif) ligand 8** | **403850** | **31.5 ± 13.7^a^** | **14.9 ± 1.8^a^** | | **125 ± 28.3^b^** | **90.3 ± 15.8^b^** | | **0.007** | |
| ***CXCL10*** | **C-X-C motif chemokine ligand 10 (IP-10)** | **478432** | **15.9 ± 3.2^a^** | **22 ± 1^a^** | | **65.6 ± 17.5^b^** | **83 ± 8.8^b^** | | **0.003** | |
| *FFAR2* | Free fatty acid receptor 2 (G-protein receptor GPR)43) | 484580 | 0 ± 0 | 0 ± 0 | | 3.8 ± 3.8 | 0 ± 0 | | 0.441 | |
| *FFAR3* | Free fatty acid receptor 3 (GPR41) | 612659 | 3.6 ± 3.6 | 0 ± 0 | | 7.9 ± 4 | 4.3 ± 4.3 | | 0.491 | |
| *FOXP3* | Forkhead box P3 | 491876 | 0 ± 0 | 3.7 ± 3.7 | | 0 ± 0 | 0 ± 0 | | 0.441 | |
| *GJA1* | Gap junction protein alpha 1 | 403418 | 15.9 ± 8.1 | 11.4 ± 5.8 | | 6.7 ± 6.7 | 12.9 ± 6.4 | | 0.813 | |
| *GJA5* | Gap junction protein alpha 5 | 483155 | 4.4 ± 4.4 | 4 ± 4 | | 0 ± 0 | 0 ± 0 | | 0.595 | |
| *HCAR2* | Hydroxycarboxylic acid receptor 2 (GPR109-A) | 486253 | 0 ± 0 | 0 ± 0 | | 0 ± 0 | 0 ± 0 | | N/A | |
| *HSP90B1* | Heat shock protein 90 beta family member 1 | 404019 | 51.1 ± 10.1 | 31.2 ± 3.6 | | 43.1 ± 13.9 | 44.5 ± 6.2 | | 0.535 | |
| *IFNG* | Interferon gamma | 403801 | 22 ± 6.1 | 30.7 ± 7.1 | | 18.9 ± 0.8 | 20.9 ± 6.7 | | 0.525 | |
| *IL1B* | Interleukin-1 beta | 403974 | 0 ± 0 | 0 ± 0 | | 0 ± 0 | 0 ± 0 | | N/A | |
| *IL10* | Interleukin-10 | 403628 | 18.2 ± 4.3 | 9.6 ± 4.8 | | 5.2 ± 5.2 | 16.3 ± 8.2 | | 0.425 | |
| *IL12A* | Interleukin-12A | 403977 | 16.5 ± 2.9 | 11.5 ± 5.9 | | 9.4 ± 4.7 | 14.4 ± 1 | | 0.638 | |
| *IL13* | Interleukin-13 | 442990 | 0 ± 0 | 0 ± 0 | | 0 ± 0 | 0 ± 0 | | N/A | |
| *IL18* | Interleukin-18 | 403796 | 4 ± 4 | 11 ± 5.5 | | 0 ± 0 | 8.5 ± 4.2 | | 0.295 | |
| *IL2* | Interleukin-2 | 403989 | 0 ± 0 | 3.6 ± 3.6 | | 0 ± 0 | 3.7 ± 3.7 | | 0.595 | |
| *IL4* | Interleukin-4 | 403785 | 0 ± 0 | 0 ± 0 | | 0 ± 0 | 0 ± 0 | | N/A | |
| *IL6* | Interleukin-6 | 403985 | 0 ± 0 | 0 ± 0 | | 0 ± 0 | 0 ± 0 | | N/A | |
| *IRAK1* | Interleukin-1 receptor-associated kinase 1 | 492247 | 0 ± 0 | 0 ± 0 | | 0 ± 0 | 0 ± 0 | | N/A | |
| *IRAK2* | Interleukin-1 receptor-associated kinase 2 | 484657 | 0 ± 0 | 0 ± 0 | | 0 ± 0 | 3.7 ± 3.7 | | 0.441 | |
| *IRAK3* | Interleukin-1 receptor-associated kinase 3 | 481150 | 0 ± 0 | 0 ± 0 | | 0 ± 0 | 0 ± 0 | | N/A | |
| *IRAK4* | Interleukin-1 receptor-associated kinase 4 | 486601 | 0 ± 0 | 0 ± 0 | | 0 ± 0 | 0 ± 0 | | N/A | |
| *LOC485869* | Lipopolysaccharide binding protein | 485869 | 0 ± 0 | 0 ± 0 | | 0 ± 0 | 0 ± 0 | | N/A | |
| *LYZ* | Lysozyme | 474442 | 0 ± 0 | 0 ± 0 | | 0 ± 0 | 0 ± 0 | | N/A | |
| *MUC1* | Mucin 1, cell surface associated | 448784 | 0 ± 0 | 0 ± 0 | | 0 ± 0 | 0 ± 0 | | N/A | |
| *MUC2* | Mucin 2, oligomeric mucus/gel-forming | 119864303 | 0 ± 0 | 0 ± 0 | | 0 ± 0 | 0 ± 0 | | N/A | |
| *MYD88* | Myeloid differentiation primary response 88 | 477024 | 5.3 ± 5.3 | 0 ± 0 | | 10.3 ± 5.4 | 4.3 ± 4.3 | | 0.469 | |
| *NFKB1* | Nuclear factor kappa B subunit 1 | 442859 | 17.6 ± 2 | 18.5 ± 4.2 | | 17.8 ± 4.1 | 17.2 ± 3.5 | | 0.995 | |
| *NOD1* | Nucleotide-binding oligomerization domain containing 1 | 482382 | 3.8 ± 3.8 | 3.7 ± 3.7 | | 0 ± 0 | 13 ± 6.7 | | 0.254 | |
| *NOD2* | Nucleotide-binding oligomerization domain containing 2 | 487286 | 0 ± 0 | 0 ± 0 | | 0 ± 0 | 0 ± 0 | | N/A | |
| *OCLN* | Occludin | 403844 | 47 ± 12.9 | 23.7 ± 0.3 | | 31.8 ± 11.5 | 37.6 ± 10.1 | | 0.458 | |
| *PDCD1* | Programmed cell death 1 | 486213 | 13.6 ± 7.1 | 18 ± 5.2 | | 14.7 ± 0.6 | 15.8 ± 2.6 | | 0.914 | |
| *PRKCA* | Protein kinase C alpha | 490904 | 4.9 ± 4.9 | 0 ± 0 | | 3.8 ± 3.8 | 5.7 ± 5.7 | | 0.784 | |
| *SAA1* | Serum amyloid A1 | 6288 | 11.9 ± 6 | 6.3 ± 6.3 | | 15.2 ± 0.4 | 8.1 ± 4.1 | | 0.587 | |
| *STAT3* | Signal transducer and activator of transcription 3 | 490967 | 13.9 ± 6.9 | 5.6 ± 5.6 | | 10 ± 5.1 | 14.5 ± 1.8 | | 0.626 | |
| *TIRAP* | Toll-interleukin 1 receptor (TIR) domain containing adaptor protein | 609544 | 0 ± 0 | 3.7 ± 3.7 | | 0 ± 0 | 3.8 ± 3.8 | | 0.596 | |
| *TLR1* | Toll-like receptor 1 | 488834 | 21.9 ± 4.9 | 20 ± 3.4 | 15.8 ± 2.4 | | 14.8 ± 7.5 | 0.712 | |  |
| *TLR10* | Toll-like receptor 10 | 100379585 | 27.3 ± 5 | 25.2 ± 2.4 | 22.4 ± 6 | | 37.8 ± 8.3 | 0.335 | |  |
| *TLR2* | Toll-like receptor 2 | 448807 | 13.9 ± 0.5 | 4.5 ± 4.5 | 0 ± 0 | | 4.3 ± 4.3 | 0.069 | |  |
| *TLR3* | Toll-like receptor 3 | 482905 | 0 ± 0 | 0 ± 0 | 0 ± 0 | | 0 ± 0 | N/A | |  |
| *TLR4* | Toll-like receptor 4 | 403417 | 4.9 ± 4.9 | 7 ± 7 | 6.7 ± 6.7 | | 0 ± 0 | 0.787 | |  |
| *TLR5* | Toll-like receptor 5 | 488605 | 13.1 ± 1.5 | 8.6 ± 4.3 | 9.1 ± 4.7 | | 11.3 ± 5.8 | 0.88 | |  |
| *TLR6* | Toll-like receptor 6 | 111089957 | 16.5 ± 2.2 | 20.4 ± 4.1 | 15.5 ± 8.7 | | 12.8 ± 6.5 | 0.839 | |  |
| *TLR7* | Toll-like receptor 7 | 491743 | 11.7 ± 6.6 | 0 ± 0 | 11.1 ± 5.9 | | 13.3 ± 1.4 | 0.218 | |  |
| *TLR8* | Toll-like receptor 8 | 100684828 | 0 ± 0 | 0 ± 0 | 0 ± 0 | | 3.7 ± 3.7 | 0.441 | |  |
| *TLR9* | Toll-like receptor 9 | 403502 | 4 ± 4 | 5.1 ± 5.1 | 0 ± 0 | | 5.4 ± 5.4 | 0.791 | |  |
| *TJP1* | Tight junction protein 1 (zonula occludin (ZO)-1) | 403752 | 0 ± 0 | 4.2 ± 4.2 | 4.3 ± 4.3 | | 3.7 ± 3.7 | 0.8 | |  |
| *TJP2* | Tight junction protein 2 (ZO-2) | 403854 | 8 ± 4.1 | 4.9 ± 4.9 | 4.7 ± 4.7 | | 4.3 ± 4.3 | 0.932 | |  |
| *TNF* | Tumour necrosis factor (TNF) | 403922 | 0 ± 0 | 0 ± 0 | 0 ± 0 | | 0 ± 0 | N/A | |  |
| *TRAF6* | TNF receptor-associated factor 6 | 100688110 | 9.7 ± 4.9 | 9.6 ± 5 | 10.7 ± 5.3 | | 18.4 ± 2.6 | 0.515 | |  |
| ***Housekeeping Genes*** | | |  |  |  |  |  |  |  |  |
| *SLC5A1* | Solute carrier family 5 member 1 | 492299 |  |  |  |  |  |  |  |  |
| *SLC3A1* | Solute carrier family 3 member 1 | 403700 |  |  |  |  |  |  |  |  |
| *FABP1* | Fatty acid binding protein 1 | 403619 |  |  |  |  |  |  |  |  |
| *FABP2* | Fatty acid binding protein 2 | 119867213 |  |  |  |  |  |  |  |  |
| *ABCB1* | ATP binding cassette subfamily B member 1 | 403879 |  |  |  |  |  |  |  |  |

**Supplementary Table S2:** Cytokine and chemokine concentrations in pg/mL of cIEC samples collected 8 hours after a challenge with 1 mM sodium butyrate (Butyrate), 250 μg/mL lipopolysaccharides (LPS) or both 1 mM sodium butyrate and 250 μg/mL lipopolysaccharides (Combination). Samples are comprised of apical media (n = 24) and basal media (n = 24). n = 6 for each treatment. Cytokine concentrations are presented as means ± SEM. Results that do not share the same superscript letter denotes a significant difference in cytokine concentration (p < 0.05). Significant p values and associated proteins are listed in bold.

| **Protein** | **Description** | **Apical cell media** | | | | **Basal cell media** | | | | **P value** |
| --- | --- | --- | --- | --- | --- | --- | --- | --- | --- | --- |
|  |  | **Control** | **Butyrate** | **LPS** | **Combination** | **Control** | **Butyrate** | **LPS** | **Combination** |  |
| GM-CSF | Granulocyte-macrophage colony-stimulating factor | 0.0 ± 0.0 | 0.0 ± 0.0 | 0.1 ± 0.1 | 0.0 ± 0.0 | 0.7 ± 0.5 | 0.0 ± 0.0 | 1.3 ± 1.0 | 0.0 ± 0.0 | 0.364 |
| IFN-γ | Interferon gamma | 0.0 ± 0.0 | 0.0 ± 0.0 | 0.0 ± 0.0 | 0.0 ± 0.0 | 0.4 ± 0.4 | 0.0 ± 0.0 | 1.0 ± 0.6 | 0.0 ± 0.0 | 0.213 |
| **KC-like** | **Keratinocyte chemotactic-like** | **6.6 ± 0.3^a^** | **3.4 ± 0.1^a^** | **47.1 ± 2.4^b^** | **48.6 ± 3.8^b^** | **2.7 ± 0.2^a^** | **1.1 ± 0.7^a^** | **49.8 ± 5.8^b^** | **0.0 ± 0.0^a^** | **< 0.001** |
| **IP-10/CCL10** | **Interferon gamma-induced protein 10** | **0.0 ± 0.0^a^** | **0.0 ± 0.0^a^** | **0.0 ± 0.0^a^** | **0.0 ± 0.0^a^** | **0.0 ± 0.0^a^** | **2.5 ± 0.8b** | **0.0 ± 0.0^a^** | **4.6 ± 0.1^c^** | **< 0.001** |
| IL-2 | Interleukin 2 | 0.0 ± 0.0 | 0.0 ± 0.0 | 0.0 ± 0.0 | 0.0 ± 0.0 | 4.3 ± 4.3 | 0.0 ± 0.0 | 8.8 ± 4.7 | 0.0 ± 0.0 | 0.177 |
| IL-6 | Interleukin 6 | 0.0 ± 0.0 | 0.0 ± 0.0 | 0.0 ± 0.0 | 0.0 ± 0.0 | 2.7 ± 2.6 | 0.0 ± 0.0 | 6.2 ± 3.3 | 0.0 ± 0.0 | 0.135 |
| IL-7 | Interleukin 7 | 0.0 ± 0.0 | 0.0 ± 0.0 | 0.0 ± 0.0 | 0.0 ± 0.0 | 5.2 ± 5.2 | 0.0 ± 0.0 | 12.1 ± 6.8 | 0.0 ± 0.0 | 0.166 |
| **IL-8** | **Interleukin 8** | **63.1 ± 22.6^a^** | **0.0 ± 0.0^a^** | **481.6 ± 25.2^b^** | **414.0 ± 18.0^b^** | **28.7 ± 28.7^a^** | **0.0 ± 0.0^a^** | **304.0 ± 24.0^c^** | **163.5 ± 51.3^d^** | **< 0.001** |
| IL-10 | Interleukin 10 | 0.0 ± 0.0 | 0.0 ± 0.0 | 0.0 ± 0.0 | 0.0 ± 0.0 | 0.0 ± 0.0 | 0.0 ± 0.0 | 0.0 ± 0.0 | 0.0 ± 0.0 | N/A |
| IL-15 | Interleukin 15 | 0.2 ± 0.2 | 0.0 ± 0.0 | 0.0 ± 0.0 | 0.0 ± 0.0 | 5.0 ± 4.5 | 0.0 ± 0.0 | 6.6 ± 5.1 | 0.0 ± 0.0 | 0.414 |
| IL-18 | Interleukin 18 | 0.0 ± 0.0 | 0.0 ± 0.0 | 0.0 ± 0.0 | 0.0 ± 0.0 | 0.8 ± 0.7 | 0.0 ± 0.0 | 2.2 ± 1.1 | 0.0 ± 0.0 | 0.064 |
| **MCP-1/CCL2** | **monocyte chemoattractant protein-1** | **1338.8 ± 36.5^a^** | **855.2 ± 86.8^b^** | **1529.6 ± 44.8^a^** | **1427.1 ± 17.3^a^** | **350.9 ± 27.6^c^** | **613.6 ± 25.6^d^** | **1111.6 ± 29.1^e^** | **1119.5 ± 19.8^e^** | **< 0.001** |
| TNF-α | Tumour necrosis factor alpha | 0.0 ± 0.0 | 0.0 ± 0.0 | 0.0 ± 0.0 | 0.0 ± 0.0 | 1.6 ± 1.6 | 0.0 ± 0.0 | 3.8 ± 2.6 | 0.0 ± 0.0 | 0.241 |
